# Regenerative endodontic therapy in immature teeth using photobiomodulation and photodynamic therapy; a histomorphological study in canine model

**DOI:** 10.1186/s12903-024-05189-3

**Published:** 2024-11-23

**Authors:** Eman M. Fouad, Mervat I. Fawzy, Ali M. Saafan, Maha A. Elhousiny

**Affiliations:** 1 Division of Endodontics, Faculty of Oral and Dental Surgery, Misr University for Science and Technology, Giza, Egypt; 2 Professor of Endodontics Department, Faculty of Dental Medicine for Girls, Al-Azhar University, Cairo, Egypt; 3 Professor of Dental Laser Applications, NILES, Cairo University, Giza, Egypt; 4 Associate professor of Endodontics, Faculty of Dental Medicine for Girls, Al-Azhar University, Cairo, Egypt

**Keywords:** Regenerative endodontics, Cell homing, Laser, Photobiomodulation, Biostimulation, Photodynamic therapy, ROS, Root canal disinfection

## Abstract

**Background:**

Regenerative endodontic therapy (RET) is still coming up short to demonstrate histological evidence for true regeneration with clinically feasible protocol of cell homing in single visit approach.

**Aim:**

The aim of the present study is to evaluate the regenerative potential of photobiomodulation (PBM) on RET in immature roots when photodynamic therapy (PDT) protocol is implemented for root canal disinfection in canine model.

**Materials and methods:**

Seventy-two root canals were recruited, with sixty assigned to experimental groups and twelve to positive and negative controls. Following the induction of pulp necrosis and apical periodontitis, the roots were divided into two experimental groups: Group I received RET followed by PBM (seven sessions with an 808 nm diode laser at 300 mW for 90 s), and Group II received RET without PBM. Follow-ups were conducted at 1, 2, and 3 months (subgroups A, B, and C respectively). Qualitative and quantitative assessment was carried out histologically. All data were statistically analyzed with the Mann-Whitney U test and Bonferroni’s adjustment, as well as Chi square test.

**Results:**

The newly formed hard tissue highly resembled true dentine where the dentinal tubules looked well organized lined by poly layers palisading pattern of rounded odontoblast-like cells with cytoplasmic processes extending through the predentine layer. GI exhibited statistically significantly higher scores of vital tissue infiltration and hard tissue deposition in subgroups A and B (*P* ≤ 0.05). The inflammatory cells scores were significantly lower in GI than in GII at all time intervals. However, no significance could be detected regarding apical closure.

**Conclusion:**

The disinfection protocol of PDT and subsequent irradiation with low power laser in PBM protocol pose a promising potential for regenerative endodontics in immature teeth.

## Introduction

Regenerative endodontic therapy is an innovative approach that uses tissue engineering techniques to restore pulp functionality [1]. This biologically based treatment yields positive clinical outcomes and supports the continued maturation of roots. RET is particularly advantageous for treating necrotic immature permanent teeth, offering a viable alternative to single visit apexification using MTA apical plugs [2].

The core foundation of pulp regeneration lies in the triad of stem/progenitor cells, scaffolds, and signaling molecules. The primary strategy for RET is the recruitment of endogenous stem cells, known as cell homing, as cell-based RET is not yet routine due to ethical and technical challenges [3].

Effective root canal disinfection is crucial for the success of regenerative endodontics [4]. A delicate balance must be achieved between thorough disinfection and the modification of the dentine matrix to release growth factors and preserve the vitality of progenitor cells. This balance enhances the microenvironment, facilitating stem cell migration and repopulation [5]. Current disinfection protocols fall short for eliminating intracanal microorganisms, as demonstrated in simulated regeneration models [6]. While single-visit RET has shown some promise, its efficacy remains inconclusive [7]. Therefore, developing alternative disinfection strategies with sufficient antimicrobial efficacy, biocompatibility, and regenerative potential is critical in this context.

Laser disinfection has emerged as a promising adjunct or alternative to traditional root canal disinfection methods. A recent study has shown it to be as effective as standard antibiotic paste protocols used in RET [8]. Moreover, photodynamic therapy (PDT), which uses a light-activated photosensitizer to produce reactive oxygen species, could enhance root canal disinfection in the literature. Adjuvant PDT achieved complete bacterial elimination in over half of the treated root canals [9].

Photobiomodulation (PBM), which involves the use of low-power laser energy (typically up to 500 mW), has demonstrated synergistic effects in enhancing stem cell proliferation and functionality [10]. PBM has been described as the fourth dimension in pulp regenerative efforts due to its positive impact on cell viability, proliferation, and differentiation, particularly under stressed conditions [11]. PBM enhances cellular functions by activating cytochrome C oxidase, which triggers redox states and cellular reactive oxygen species (ROS) production [12]. This has led to significant in vivo improvements, positioning PBM as a potential adjunct in pulp regenerative therapies [13].

The outcomes of regenerative processes should be evaluated based on histopathological findings to clarify the nature of the newly formed tissue and assess the restoration of tissue functionality. Translational animal studies are invaluable in this context, as such evidence cannot be obtained through clinical trials due to ethical constraints [14]. This evidence is essential to distinguish true regeneration from mere restorative repair [15].

A review of the literature reveals a lack of histological evidence on the use of PDT for root canal disinfection in RET. This gap necessitates further investigation, as PDT could offer a promising protocol to balance effective root canal disinfection with microenvironment enhancement.

Additionally, the impact of PBM on the regenerative potential of root maturation following PDT disinfection remains underexplored and warrants further investigation. Therefore, this study aims to histologically evaluate the effect of PBM on the response of immature teeth with necrotic pulp and apical periodontitis in dogs undergoing RET with PDT disinfection. The null hypothesis of this study is that there is no significant difference in the histomorphological outcomes of RET in immature teeth with necrotic pulp and apical periodontitis in dogs when PBM is applied after PDT for root canal disinfection compared to RET without PBM.

## Materials and methods

The research ethics committee at the Faculty of Dental Medicine for Girls, Al-Azhar University, approved this study (REC-EN-21-09 B). To ensure the welfare of the animals, the study was conducted in accordance with the ARRIVE guidelines [16] for animal research. The research protocol was registered at Preclinicaltrials.eu [PCTE0000162] and was undertaken at Governmental Veterinary Hospital (GVH) in Abbasia. The dogs were owned by the GVH, and an informed consent was obtained.

A total of three healthy, 4–6 months old beagle dogs with no sex predilection were recruited for the study based on sample size calculation on the criteria of 80% power of calculation and α level of 0.05 and meaningful difference in previous studies totaling 72 root canals [7, 17].

Experimental root canals (60 roots) in 30 premolar teeth were assigned to one of the two experimental groups in a split-mouth protocol,

Group I: PDT-based root canal disinfection with subsequent sessions of PBM.

Group II: PDT-based root canal disinfection without subsequent sessions of PBM.

Positive control was presented as root canals left untreated after induction of necrosis and periapical infection while negative control reflected healthy teeth with normal tooth development. Dogs were randomly allocated by means of closed envelopes according to the post-treatment evaluation periods, one, two, or three months classified into three subgroups (A, B, and C) respectively.

### Induction of periapical lesions

Dogs were premedicated with intravenous atropine sulfate (Atropine^®^ CID Co, Egypt) at a dose of 0.05 mg/kg and diazepam (Neuril^®^ Memphis Co, Egypt) at 1 mg/kg. Anesthesia was induced using intravenous ketamine HCl (Ketamine^®^ Sigmatec Co, Egypt) at 10 mg/kg and xylazine (Xylaject^®^ Adwia Co, Egypt) at 1 mg/kg. To maintain the depth of anesthesia, thiopental sodium 2.5% (Thiopental Sodium^®^ Sandoz, Austria) was administered intravenously at 25 mg/kg.

Under aseptic conditions, a #2 round bur attached to a high-speed handpiece was used to access the pulp chambers. The pulp tissue within the root canals was then disrupted using a stainless-steel endodontic hand file (#30). The access cavities were temporarily sealed with cotton pellets and left undisturbed for three weeks. For pain management, the animals received carprofen (Rimadyl^®^ tablets, Zoetis, USA) at a dosage of 4.4 mg/kg, administered once daily for one week.

### Root canal disinfection and regenerative procedure

Infected teeth were re-entered under a septic conditions achieved with cotton roll isolation and surface disinfection with 0.12% chlorhexidine (Paroex, Germany) and tincture of iodine under the previously indicated anesthetic regimen. The teeth were passively irrigated with 10 mL of 1.5% NaOCl (ADCO, Egypt) for around 5 min followed by 10 ml saline irrigation using a side-vented needle 1 mm short of the apex. root canals were not instrumented.

Root canals were disinfected by PDT. Canals were inoculated with 50 µM methylene blue (Sigma, USA) dye (MB) to fill the entire pulp space, left undisturbed for one minute and then activated by 660 nm diode laser (Pioon S1, China) with output power 100 mW for 90 s with the aid of fiber optic #220 µm introduced 1 mm short of the apex and moved up and down the canal in spiral mode, giving total energy of 9 joules per root canal. The protocol of RET was subsequently initiated up to the recommendations of the American Associations of Endodontics (AAE) [18]. Root canals were irrigated with 10 ml of saline followed by 10 ml 17% EDTA (Grace, Egypt) irrigation and finally rinsed with 10 ml of saline. The regenerative procedure was initiated immediately on the same visit. The entire canal was filled with blood due to over-instrumentation with the purpose of blood clot formation.

A coronal plug 3–4 mm thick of MTA was placed over the freshly formed blood clot (Bio MTA, Cerkamed, Poland) afterward, the teeth were permanently restored with glass ionomer restoration (GC Fuji, GC America, Alsip, IL).

### Laser photobiomodulation

In the experimental group G I, low-power diode laser radiation (808 nm wavelength) with an output power of 300 mW was carried on with a circular irradiating tip (6 mm radius). For 90 s, the buccal side, opposite to the periapical area and root tips, received energy at a fluency of 27 J/cm^2^ in continuous wave, non-contact mode. This regime was initiated directly after the RET and continued every other day for a total of seven sessions.

### Histologic evaluation

At the end of each follow-up period, the dog was euthanized by an overdose of anesthetic solution (20 mL of thiopental sodium 5% solution), jaws were separated and bone segments including the experimental and control teeth were resected. Blocks were fixed by 10% buffered formalin solution at the ratio of fixative and tooth mass 1:5 respectively and kept for about 2 weeks. Blocks were then decalcified using a mixture of formic acid and sodium citrate solution which was renewed every other day for four months. Specimens were dehydrated by ethyl alcohol, embedded in paraffin blocks, and consequently sectioned in buccolingual sections of 5 μm thickness and stained by hematoxylin and eosin dye. Sections were examined under a light microscope at X40 and X100 magnifications. Two independent blinded investigators examined all the specimens to reduce bias, Inter-examiner reliability was verified using Kappa statistics (Cohen kappa ≥ 0.86). The histologic evaluation required both qualitative and quantitative assessments of the newly formed tissues. The qualitative analysis concentrated on the characteristics of the regenerated soft and hard tissues, while the quantitative analysis used a structured scoring system to objectively measure the extent of tissue formation. The evaluation followed these parameters:

1. Evaluation of vital tissue ingrowth within the canal space was based on previously established methods [8, 19], utilizing a scoring system to measure the extent of tissue presence. The scoring system is as follows: score (0) indicates no tissue ingrowth within the canal space; score (1) represents tissue ingrowth in the apical third of the canal; score (2) reflects tissue presence extending into the middle third; and score (3) denotes tissue ingrowth in the cervical third of the canal.
2. Evaluation of new hard tissue formation was conducted following the methodology outlined in previous studies [8, 19–21]. The assessment was based on the following scoring system: score (0) indicated the absence of any new hard tissue formation, score (1) represented partial formation of hard tissue, and score (2) indicated complete formation of new hard tissue.
3. Inflammation (Intensity of inflammatory cell infiltrate) was assessed using a scoring system based on previously established methods [19, 21]. The system is as follows: score 0 indicates the absence of or very few inflammatory cells, score 1 (mild) indicates fewer than 10 cells on average, score 2 (moderate) represents an average of 10 to 25 cells, and score 3 (severe) indicates the presence of more than 25 cells.
4. Evaluation of apical closure was conducted based on previous studies [19, 20]. The scoring system is reviewed as follows: score (0) was assigned when apical closure was not observed, while score (1) indicated evidence of apical closure.

### Statistical analysis

Data were presented as median and range values. Normality was assessed using the Kolmogorov-Smirnov and Shapiro-Wilk tests. Histological scores were treated as non-parametric data. The Mann-Whitney U test and Bonferroni’s adjustment were used for significant pairwise comparisons. Apical closure data were presented as frequencies and percentages, and the Chi-square test was used for comparison. Statistical analysis was performed using IBM SPSS Statistics Version 20 for Windows (IBM^®^, NY, USA).

## Results

### Group I: (RET with PBM)

**Fig. 1:**
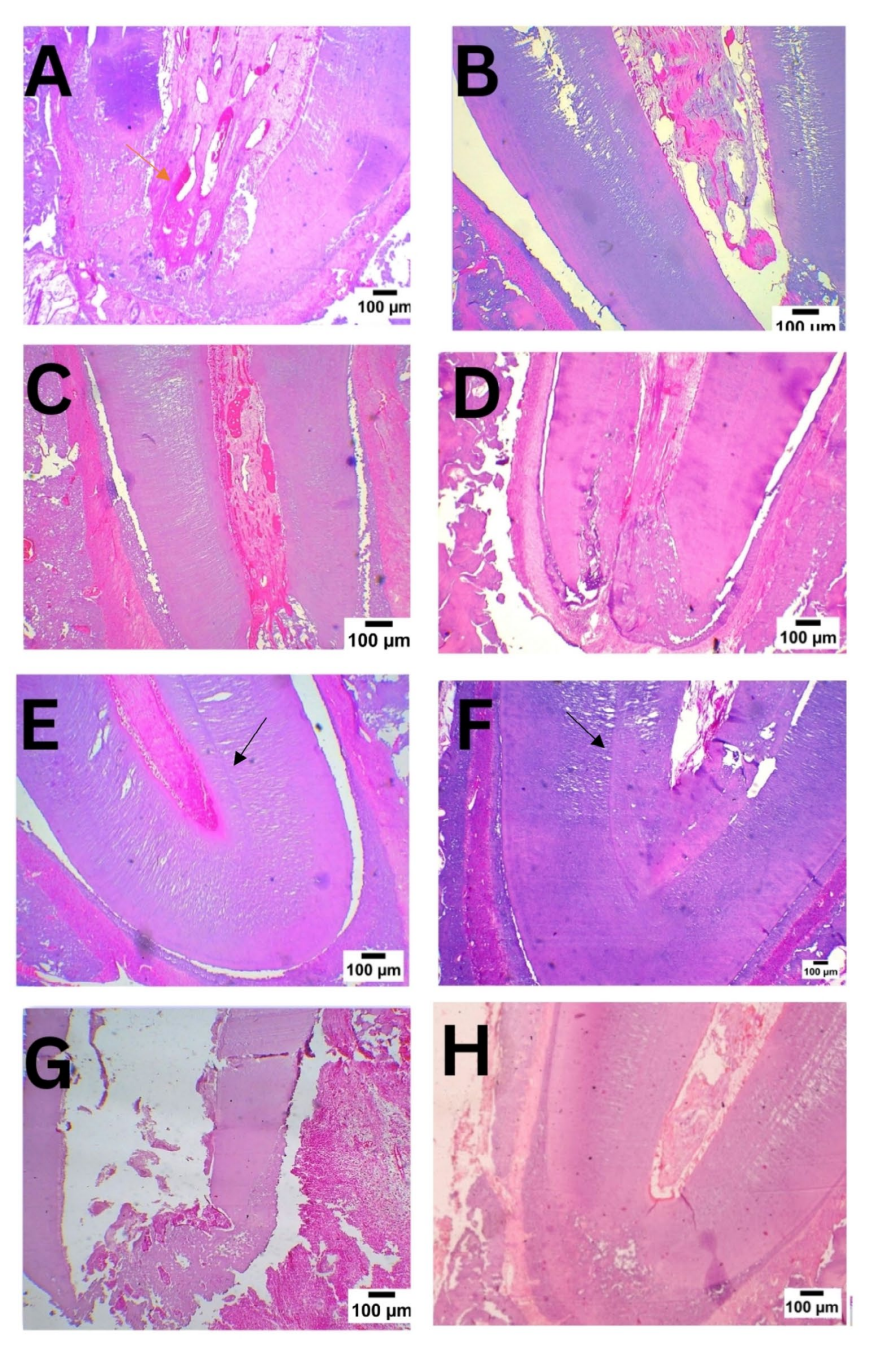
Photomicrograph of samples in different subgroups (H&E 100X) showing vital tissue infiltration, angiogenesis (red arrow) and open apex at first month in GI A in and GII A in Fig. **1A** and **B** respectively. Subgroups B demonstrate partial dentine deposition and signs of apical narrowing in Fig. **1C** and **D** respectively. Subgroup C demonstrates evident apical closure. The newly formed hard tissue is separated from the old dentine by demarcation line (black arrow) in Fig. **1E** and **F** respectively. The newly deposited mineralized tissue resembles the architecture of dentinal tubules and a definite predentine layer, particularly in GI subgroup C (Fig. **1E**). At the third month, Fig. **1G** represents positive control whereas Fig. **1H** represents negative control

**Fig. 2:**
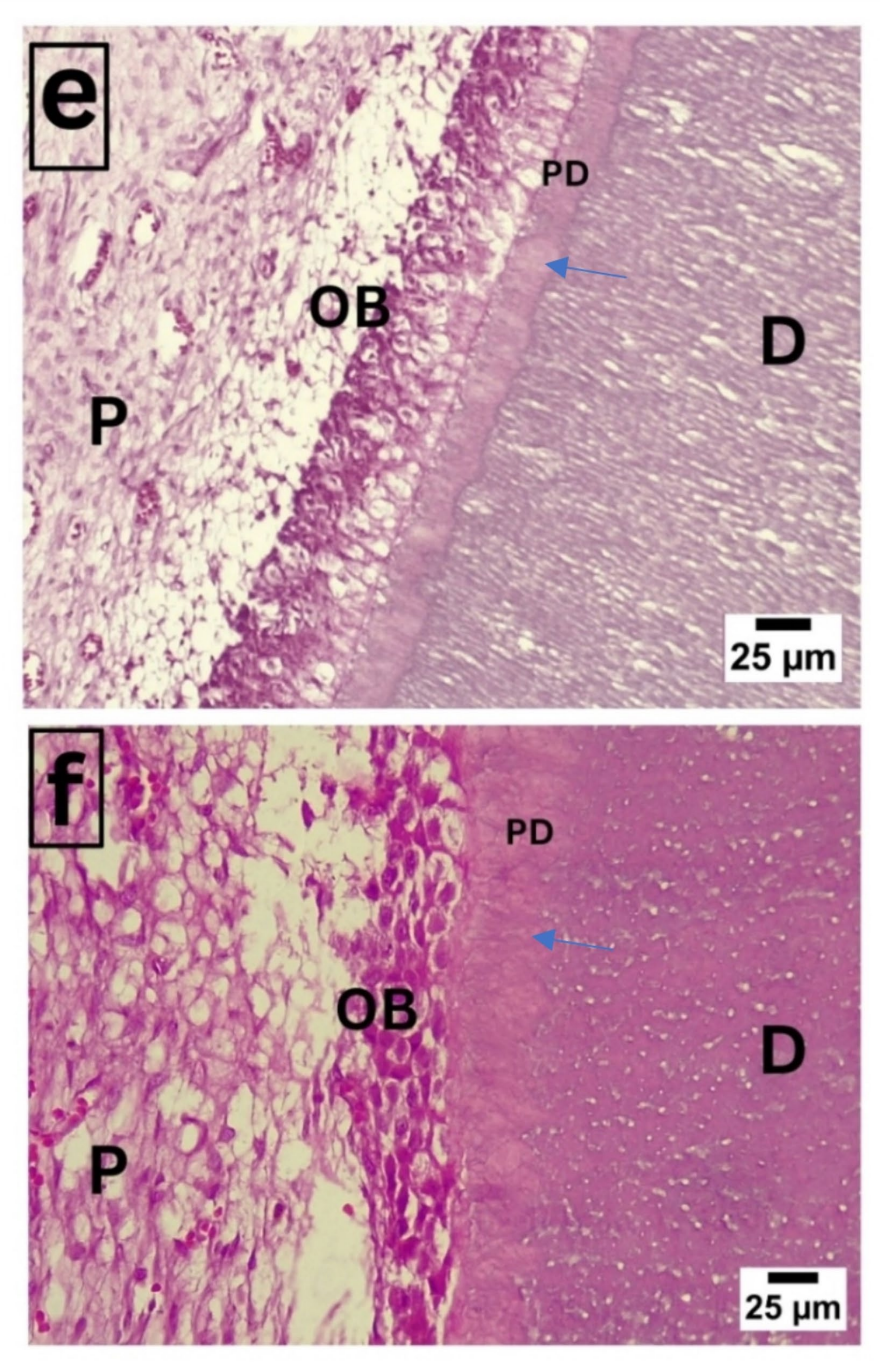
Photomicrograph of samples in GI and GII at third month (H&E 400X) showing odontoblast like cells lining the newly deposited hard tissue in more elongated cells in palisading pattern in Fig. **2e** and the cytoplasmic processes extending through a definite predentine layer (blue arrow). Abbreviations: P; pulplike tissue, **D**; dentine like hard tissue, OB; odontoblast-like cells, PD; predentine

In subgroup A, vital tissue infiltration was observed filling the canal, progressing to fill the entire root canal space by the second time interval in subgroups B and C as shown in Fig. 1A and C, and 1E respectively. Odontoblast-like cells were seen lining the newly formed, well-organized dentine-like mineralized tissue as shown in Fig. 2e, with an apparent increase in vascularity.

The newly formed hard tissue ranged from minimal formation in subgroup A to complete formation in subgroup C. The newly formed tissue closely resembled true dentine, with well-organized dentinal tubules lined by a multilayered palisading pattern of rounded odontoblast-like cells, their cytoplasmic processes extending through the predentine layer as demonstrated in Fig. 1e. While the apex remained open in subgroup A, signs of apical closure were observed in subgroup B and became more pronounced in subgroup C as presented in Fig. 1E.

### Group II: (RET without PBM)

In subgroup A, initial vital tissue infiltration was noted towards the apical third of the canal as shown in Fig. 1B, which extended coronally to fill the entire root canal space by the third month as presented in Fig. 1F. The extent of vital tissue infiltration was significantly lower in Group II compared to Group I in subgroups A and B, but no significant difference was found in subgroup C as demonstrated in Table 1.

No newly formed hard tissue was observed in subgroup A. However, abundant hard tissue deposition resembling dentine, though less organized, was noted in subgroup C as shown in Fig. 1f. A statistically significant difference in hard tissue deposition was found between the groups in subgroups A and B, but not in subgroup C as demonstrated in Table 1.

By the third time interval in subgroup C, the apex, which remained open in subgroup A, showed signs of closure as shown in Fig. 1B and D, and 1F. No significant difference was detected between the groups regarding apical closure, as indicated in Table 2.

Mild to moderate inflammation was observed near the root apex in subgroups A and B, which appeared milder in subgroup C when PBM was applied in Group I. At all-time intervals, inflammation scores were significantly lower in the PBM treatment group. Roots in the positive control group showed a consistently open apex with intense inflammation and minimal intracanal vital tissue ingrowth Fig. 1G. In contrast, roots in the negative control group showed a closed apex with normal dentine architecture and pulp histological configuration Fig. 1H.

**Table 1:** Median [minimum-maximum] of scores of vital tissue infiltration, hard tissue formation and inflammatory cells in both groups

Outcome/Subgroups	G I (with PBM)	G II (without PBM)	*P* value
**Vital tissue infiltration**			
Subgroup A	2 [1–3]	1[0–2]	0.020*
Subgroup B	2.5 [2–3]	2 [1–3]	0.042*
Subgroup C	3 [2–3]	3[2–3]	0.131
**Hard tissue formation**			
Subgroup A	1[0–1]	0[0–1]	0.028*
Subgroup B	2[1–2]	1 [0–2]	0.018*
Subgroup C	2 [2–2]	2[1–2]	0.067
**Inflammatory cells score.**			
Subgroup A	2[1–3]	3[2–3]	0.044*
Subgroup B	1[0–3]	2[1–3]	0.029*
Subgroup C	1[0–1]	1[1–2]	0.010*

* Significant difference at *P* ≤ 0.05

**Table 2:** Percent of roots with evident apical closure in each subgroup for both experimental groups

Apical closure	G I (with PBM)	G II (without PBM)	*P* value
Subgroup A	20.00%	20.00%	1
Subgroup B	70.00%	60.00%	0.5
Subgroup C	90.00%	80.00%	0.531

* Significant difference at *P* ≤ 0.05

## Discussion

Regenerative endodontics integrates tissue engineering principles into endodontic practice. With the growing demand for pulp regeneration over repair, it is essential to gather more histological evidence to characterize the newly formed soft and hard tissues.

The use of suitable animal models, like beagle dogs, is justified, especially given the limited evidence on the combined effects of photodynamic therapy (PDT) and photobiomodulation (PBM) in RET. The anatomy and physiology of dog teeth closely resemble those of human teeth, and their premolars are of an appropriate size for experimental procedures [22]. Consequently, they have been extensively used in regenerative research [23].

In this study, a blood clot was used as scaffold in the regenerative procedure. This scaffold is reliable, easy to apply, and demonstrated clinical outcomes comparable to commonly used scaffolds in RET [24]. Mechanical instrumentation of the root canal was avoided to prevent further weakening of the fragile root dentin and to preserve any remaining viable stem cells [25]. Instead, canal debridement relied solely on the chemical action of the disinfection protocol. This protocol, which included PDT and low-concentration NaOCl irrigation, was designed to achieve an optimal balance between preserving stem cell vitality and ensuring effective disinfection. The use of PDT is supported by literature demonstrating its antimicrobial and antibiofilm efficacy [26]. Current data further validate its effectiveness as comparable to standard disinfection medicaments in RET [25]. Notably, it has a stimulative effect, evidenced by increased cell viability and enhanced angiogenesis [27]. Additionally, it has been shown that activated methylene blue can reduce the rate of antibiotic-induced cell death in dental pulp stem cells [28].

To mitigate the quenching effect of MB, which can reduce reactive oxygen species (ROS) generation and the potential limitations in light penetration due to optical shielding [29], MB was used at a concentration of 50 µM coupled with laser energy. Furthermore, dental pulp cells showed reduced viability in response to higher MB concentrations in a dose-dependent manner [30].

The PBM protocol used in this study delivered an energy fluence of 27 J/cm², significantly higher than the 1–7 J/cm² range used in previous studies [11]. This increased fluence was intended to compensate for the attenuation of laser energy caused by irradiation through the buccal bone and overlying mucosa in an in vivo model, as opposed to direct irradiation of stem cells in in vitro settings.

The successful outcomes of single-visit RET in this study contrast with the low success rates reported by Botero et al. [7]. This discrepancy may be due to the enhanced disinfection achieved with PDT, compared to relying solely on NaOCl irrigation. The regenerative potential observed with the PDT protocol aligns with findings by Eldessoky et al. [8] and Cerqueira-Neto et al. [31].

The newly formed hard tissues in this study closely resembled true dentin, characterized by well-defined dentinal tubules. This architecture underscores the true regenerative nature of the process, which is rarely reported in the literature and usually involves more complex procedures [32].

The organized regenerative architecture observed in this study could be attributed to the absence of the toxic effects associated with antibiotics. Residual antibiotic pastes, despite rigorous irrigation, can impair stem cell attachment and differentiation and alter the physiological environment, thereby inactivating growth factors released from dentin [33].

Recent literature has highlighted the inductive potential of PDT on cell viability and differentiation, evidenced by the increased percentage of elongated cells in SEM [27]. Furthermore, it revealed more densely attached, greater cell-cell contact with elongated cytoplasmic process, when root samples were pretreated with PDT [34], which explains the organized appearance of the newly formed dentine like structure in the current study. On the other hand, the sporadic documentation of true dentin formation in current literature often shows reduced dentinal tubule formation [35] or disorganized, newly synthesized dentin-like structures with occasional cell entrapment [20] contrary to the organized histological findings in this study.

The application of low-level laser in Group I yielded higher scores for both intracanal vital tissue infiltration and mineralized tissue deposition compared to Group II. The mean differences were statistically significant in both the first and second months, but not the third month. This synergistic effect of PBM is consistent with Malthiery et al. [36]. Current evidence supports the increased expression of angiogenic growth factors associated with PBM [37] and the significant upregulation of odontogenic genes, including alkaline phosphatase (ALP), dentin matrix protein 1 (DMP1), and dentin sialophosphoprotein [38]. Moreover, PBM enhances the release of TGF, either alone or in conjunction with EDTA [39].

On the other hand, Pereira et al. reported no significant differences in proliferation rates or mineralized nodule production compared to controls. This discrepancy may be due to methodological differences; Pereira’s study used a single exposure, whereas the present study involved multiple sessions [40]. Additionally, PBM shows its greatest effect under stressed conditions, such as nutritional deficiency, which is typically associated with the proposed regenerative process [41].

Notably, the present findings highlight the cumulative effect of both PBM and PDT. PDT exerts a stimulative effect on stem cells through ROS production, which is relevant to mineralized extracellular matrix production and mesenchymal stem cell differentiation [27, 34]. ROS also activates TGF release in a dose-dependent manner [34]. Additionally, a previous study highlighted the stimulative effect of MB mediated biomodulation on osteoblast cells through the upregulation ALP and collagen-I expression [42].

No significant differences in apical closure were observed between experimental subgroups at any evaluation period, consistent with previous findings by Ashry et al. [43], but conflicted with El Halaby et al. who reported significant difference between the experimental groups at the second month interval [19]. These inconsistencies may result from variations in the tested interventions and the challenges commonly encountered during histological scoring and evaluation.

Regarding inflammatory cell scores, there was a statistically significant difference between the two experimental groups at all time intervals. This aligns with a previous study comparing the effects of PBM with different disinfection protocols [13]. This can be explained by the effect of low-power laser on inflammatory mediators and its inhibitory effect on pro-inflammatory cytokine expression [44].

Based on the results, the null hypothesis was rejected. To the best of our knowledge, this study is the first to explore the combined effects of PDT and PBM on RET. The findings suggest that PDT has a potential for pulp regeneration. The use of beagles, due to their physiological similarities to humans, enhances the clinical relevance of the results. These outcomes not only support the feasibility of single-visit RET but also highlight the need for further research, including immunohistological studies and clinical trials, to validate the findings and enhance treatment protocols. Additionally, 3D radiographic quantitative analysis could complement the current histological data and provide a more comprehensive assessment of RET.

## Conclusion

In conclusion, this study demonstrates the potential of laser power in both photodynamic therapy and photobiomodulation in enhancing regenerative endodontic therapy (RET), particularly for true pulp regeneration. further research is needed to confirm the potentially of this protocol, paving the way for refined regenerative endodontic treatment.

## Data Availability

The datasets used and analyzed during the current study are available from the corresponding author on reasonable request.

## Abbreviations

RET: Regenerative endodontic therapy
PDT: Photodynamic therapy
PBM: Photobiomodulation
MB: Methylene blue
TGF: Transforming growth factor
ROS: Reactive oxygen species
